# Supplementary figures and images for: Biomechanical optimization of iliolumbar fixation strategies for unilateral vertical sacral fractures: Prioritizing stability-mobility balance via finite element analysis
Source: PLoS One. 2026 Jan 13;21(1):e0339705. doi: 10.1371/journal.pone.0339705 (PMC12798994; doi:10.1371/journal.pone.0339705)

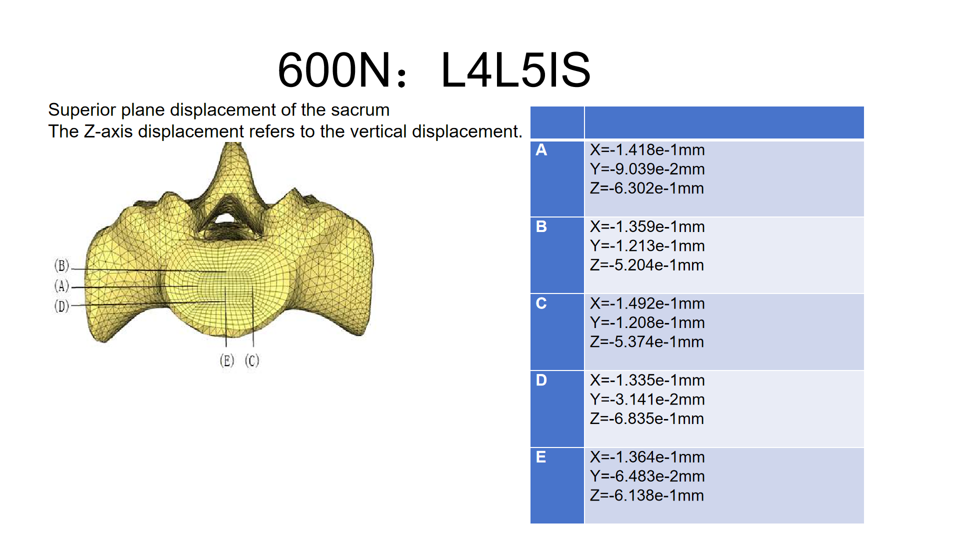

Supplement: S1 Fig — This figure displays the displacement data of 5 pre-designated measurement points on the upper surface of the sacrum under the L4L5IS fixation mode. Displacement values are presented in three spatial directions (X: anteroposterior, Y: mediolateral, Z: craniocaudal) and are derived from finite element analysis testing. (TIF) [file pone.0339705.s001.tif]

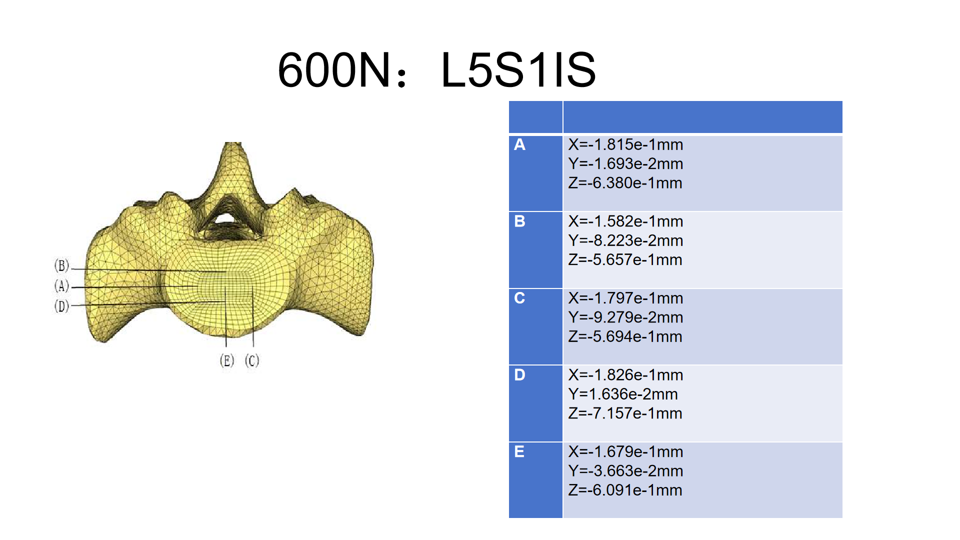

Supplement: S2 Fig — This figure displays the displacement data of 5 pre-designated measurement points on the upper surface of the sacrum under the L4L5IS fixation mode. Displacement values are presented in three spatial directions (X: anteroposterior, Y: mediolateral, Z: craniocaudal) and are derived from finite element analysis testing. (TIF) [file pone.0339705.s002.tif]

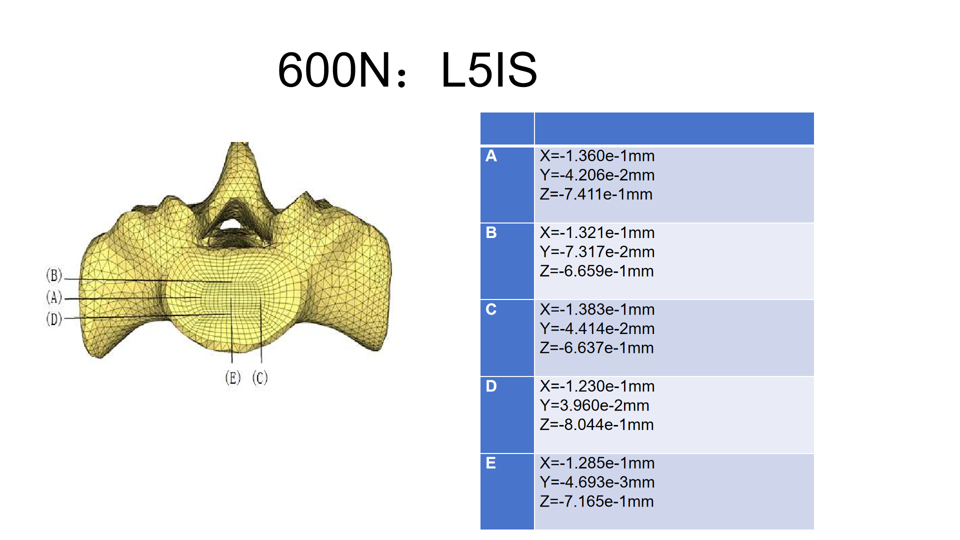

Supplement: S3 Fig — This figure displays the displacement data of 5 pre-designated measurement points on the upper surface of the sacrum under the L4L5IS fixation mode. Displacement values are presented in three spatial directions (X: anteroposterior, Y: mediolateral, Z: craniocaudal) and are derived from finite element analysis testing. (TIF) [file pone.0339705.s003.tif]

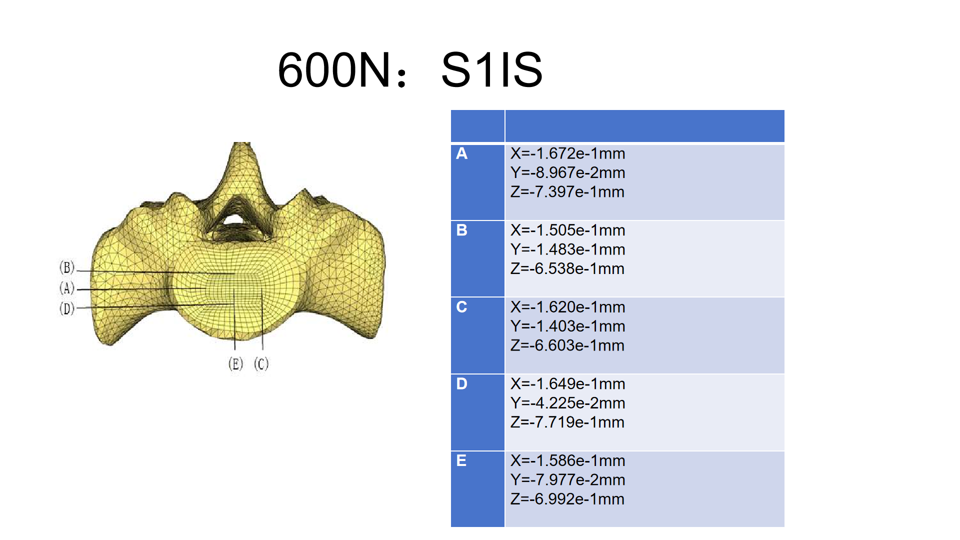

Supplement: S4 Fig — This figure displays the displacement data of 5 pre-designated measurement points on the upper surface of the sacrum under the L4L5IS fixation mode. Displacement values are presented in three spatial directions (X: anteroposterior, Y: mediolateral, Z: craniocaudal) and are derived from finite element analysis testing. (TIF) [file pone.0339705.s004.tif]
