# Supplementary material for: Biomechanical optimization of iliolumbar fixation strategies for unilateral vertical sacral fractures: Prioritizing stability-mobility balance via finite element analysis
Source: PLoS One. 2026 Jan 13;21(1):e0339705. doi: 10.1371/journal.pone.0339705 (PMC12798994; doi:10.1371/journal.pone.0339705)
Supplement: Attachment — Submitted filename: Response to Reviewers.docx [file pone.0339705.s006.docx]

Response to Reviewer#1 Comments

Response:

1.Comment :
"Make sure that you are proofreading your manuscript for uniformity as well as grammatical errors."
Response:We sincerely appreciate your comment regarding the need for careful proofreading to ensure both grammatical correctness and consistency throughout the manuscript. We have thoroughly reviewed the text to address grammatical issues and to improve uniformity in terminology, formatting, and style. We believe these revisions have significantly improved the clarity and quality of the paper. Thank you for this valuable suggestion.
2.Comment :
"Did you perform a mesh convergence study for selecting the ideal mesh size for your instrumentation as well as for the pelvis model? If yes, please provide details of the mesh convergence study.

."

Response:
We thank the reviewer for this comment. We acknowledge that a mesh convergence study is an important step for numerical simulations.

In our work, we relied on an adaptive meshing strategy to ensure accuracy. This method automatically refines the grid in geometrically complex regions and areas with high solution gradients, while using a coarser mesh in simpler domains. The maximum element size globally was controlled at 5 mm to guarantee a baseline resolution.

We believe that this adaptive approach provides a rigorous alternative to a traditional convergence study by ensuring that the mesh is sufficiently refined precisely where it is needed most. The results obtained are based on a mesh that is well-suited to capture the key phenomena of interest.

3.Comment :

“Did you perform a sensitivity study on model input parameters (e.g., material properties, loading magnitudes) ? If Yes, please provide details ”

Response:

We sincerely appreciate the reviewer for raising this important point. Conducting a sensitivity analysis is indeed crucial for thoroughly evaluating the reliability of numerical models, and we thank you for highlighting this aspect.

In the present study, all key model input parameters, including material properties and loading magnitudes, were directly adopted from well-established and widely recognized literature in the field. The values and their justifications have been explicitly stated and supported with citations in the  manuscript. Using such empirically validated values represents a common and acceptable practice in computational studies to ensure a reproducible and reliable baseline configuration.

We fully acknowledge that performing a systematic sensitivity analysis on all input parameters would constitute a significantly extensive computational effort, potentially comparable to or even exceeding the scope of the current study. Given that the primary focus of this work was toComparing biomechanical differences between different unilateral iliolumbar fixations, we have opted to designate a comprehensive parameter sensitivity study as a dedicated and separate future research endeavor.

Once again, we are grateful for your insightful suggestion, which has undoubtedly helped us improve the scholarly rigor of our work and plan for more in-depth future studies.

4.Comment :What type of elements were chosen to model the ligaments and the instrumentation ? Please elaborate

Response:

Thank you for your inquiry regarding the element types used for modeling the ligaments and instrumentation. We appreciate your attention to the technical details of our finite element setup.

For the instrumentation (internal fixation devices), we selected C3D4 elements (4-node linear tetrahedral elements) for their suitability in capturing the complex 3D stress distributions within the implants. This choice was based on two key considerations:

The C3D4 element is a 3D solid element that can accurately transmit multidirectional stresses (tensile, compressive, and shear forces) across the screws, rods, and connecting components of the fixation system, which is critical for evaluating the structural integrity of the instrumentation under physiological loads.

Given the irregular geometry of the implants (e.g., varying screw diameters, rod contours), tetrahedral elements offer flexibility in meshing while maintaining sufficient accuracy for our biomechanical analysis of stress concentrations and load transfer.

For the ligaments, we employed T3D2 elements (2-node linear 3D truss elements), which are 1-dimensional elements designed to withstand only axial tensile forces. This selection aligns with the biomechanical behavior of ligaments, which primarily resist stretching under physiological conditions rather than bending or shear. The T3D2 elements were assigned nonlinear spring properties to simulate the ligamentous response to increasing loads, ensuring that the model captures the characteristic stiffness and deformation patterns observed in biological ligaments.

5.Comment:Please elaborate on how the screw-bone interface and the screw-rod interface were simulated.

Response:Thank you for your question about our interface modeling. Both the screw-bone and screw-rod interfaces in our model were simulated using tied connections (Abaqus "Tie Constraint"), designed to reflect clinical fixation reality:

Screw-Bone Interface

We tied the screw's surface nodes to the bone's screw tunnel nodes, mimicking stable surgical fixation. This ensures no relative sliding between screw and bone under load, allowing accurate stress transfer between the implant and bone —critical for evaluating interface stability.

Screw-Rod Interface

The screw head and rod contact surfaces were tied, replicating the locked connection achieved clinically with tightening nuts. This eliminates relative movement, ensuring the entire fixation system acts as a unified structure and enabling accurate calculation of construct stiffness (a key study outcome).

Both setups align with standard orthopedic FE practices, balancing realism and computational efficiency.

6.Comment :Provide a citation/reference on why you chose the particular loading protocol.

Response:Thank you for your inquiry regarding the rationale for our loading protocol. We appreciate the opportunity to clarify the selection of the 600N vertical load in our study.

In biomechanical research on human lumbopelvic systems, a vertical load of approximately 500N is commonly used to simulate the upper body weight of an average adult in static standing [consistent with established conventions in pelvic and lumbar finite element studies]. However, when considering the transition from static standing to the initial phase of walking—a scenario highly relevant to clinical postoperative functional recovery—this upper body weight-derived force is not static. As highlighted by Winter in Biomechanics and motor control of human movement (2009, doi: 10.1002/9780470549148), the ground reaction force (and thus the axial load transmitted to the pelvis) increases by 1.1–1.3 times the static upper body weight during the early stance phase of walking. This amplification arises from the dynamic transfer of body mass and mild muscular activation required to initiate gait.

To better reflect the "functional static" loading environment that the iliolumbar fixation system may encounter in clinical practice (e.g., patients transitioning from standing to the first step of walking), we adjusted our vertical load based on this biomechanical principle. Starting from the typical 500N static upper body weight, a 1.2-fold amplification (within the 1.1–1.3 range reported by Winter) yields a load of 600N. This setting ensures our model not only simulates the basic standing condition but also accounts for the mild dynamic load increment associated with gait initiation—an important consideration for evaluating fixation stability in real-world functional scenarios.

This loading protocol thus balances physiological realism and clinical relevance, aligning with both established biomechanical theory and the practical needs of assessing our fixation strategies.

7.Comment: The conclusion section as it currently is could be improved upon by adding clinical clinical perspective with regards to instrumentation performance / failures. As it stands, it is just summation of your results.

Response:Thank you for your valuable comment on optimizing the conclusion section. We fully agree that integrating a clinical perspective on instrumentation performance and potential failures enhances the practical relevance of our results, and we have supplemented the discussion to address this.

In the added content: We linked our core findings (maximum von Mises stress of internal fixation, fracture line displacement) to clinical scenarios. We clarified that unilateral iliac-sacral fixation—mainly simulating mechanical load transfer—serves as a key alternative when sacroiliac screws are unavailable (e.g., due to limited expertise, patient-specific sacral variations, or damaged screw pathways), connecting mechanical results to real clinical needs.

We also addressed instrumentation performance and safety: We confirmed its reliability under static loading, while explicitly noting our study’s limitation—no assessment of metal stress fatigue under dynamic conditions or safety during movement. This supplements the clinical perspective on instrumentation and reminds readers of potential dynamic stress-induced risks for future practice/studies.

We believe this revision effectively responds to your suggestion, aligning the discussion more with clinical practice. Thank you again for your constructive feedback, which improved the manuscript’s completeness and clinical relevance.

8.Comment：Additionally, please address that lack of varied patient-specific factors like bone density, pelvic morphology, or age are not accounted for and a limitation of the presented study.

Response: Thank you for your valuable comment regarding the consideration of patient-specific factors. We fully agree that the lack of accounting for varied patient-specific factors (such as bone density, pelvic morphology, or age) is an important limitation of this study, and we have specifically supplemented this point in the "Limitations" section of the discussion.

As detailed in the revised Limitations section (Fifth point), we explicitly state that "patient-specific factors (e.g., bone mineral density, pelvic morphology, age) were not considered, which may limit findings’ applicability to heterogeneous real-world populations." This supplement directly addresses the limitation you pointed out, aiming to objectively reflect the constraints of the current study and provide a clear reference for the interpretation and clinical extrapolation of the research results.

We appreciate your reminder, which has further improved the completeness and rigor of our discussion on study limitations.

9.Comment：Additionally, please address that not simulating Dynamic loading activities (walking, stair climbing) is a limitation of the presented study.

Response:Thank you for your insightful comment on the simulation of dynamic loading activities. We fully concur that not simulating dynamic loading activities (e.g., walking, stair climbing) constitutes a key limitation of this study, and we have specifically incorporated this point into the "Limitations" subsection within the Discussion section of the manuscript.

This supplement directly addresses the limitation you highlighted, ensuring we objectively present the constraints of the current study’s loading simulation design within the main text’s discussion. We greatly appreciate your reminder, which has further enhanced the comprehensiveness and rigor of our analysis of study limitations.

Response to Reviewer#2 Comments

1.Comment：Lines 38–39: Please expand on the prior studies mentioned. Specify which studies have used finite element analysis, what models or fixation strategies they investigated, and their main findings. This will help highlight the novelty and distinction of your current work.

Response:

Thank you for your constructive suggestion to expand on prior studies, which helps highlight our work’s novelty. We have revised the relevant section to clarify the context of existing research and our study’s distinction, as follows:

The core focus of this study is to evaluate the effectiveness, stability, and stress distribution of internal fixation for sacral fractures under different fixation modes. Prior biomechanical studies on sacral fracture fixation (cited as [4,5,11,14,15,18]) have primarily centered on two key areas: 1) sacroiliac screw fixation and 2) triangular fixation techniques. While some of these studies employed finite element analysis (FEA) to investigate their respective models, their main focus was on comparing the biomechanical performance of sacroiliac screws and triangular fixation—with findings confirming the basic stability of these two strategies for sacral fracture fixation.

Notably, however, prior research has paid limited attention to iliolumbar fixation: existing studies related to iliolumbar fixation are mostly limited to comparative analyses with sacroiliac screws or triangular fixation, and there is a clear gap in biomechanical studies specifically investigating iliolumbar fixation’s performance (e.g., stability, stress distribution) for sacral fractures—especially in clinical scenarios where sacroiliac screw channels are damaged or medical conditions/expertise limit screw use.

To address this knowledge gap, our current study uses FEA to systematically investigate the biomechanical properties of different internal fixation models for iliolumbar fixation. This design not only differentiates our work from prior studies (which focused on other fixation strategies) but also fills the lack of targeted biomechanical data on iliolumbar fixation, ultimately aiming to provide more evidence for effective clinical fixation strategies.

We believe this revision better contextualizes prior research and highlights the novelty of our current work, as requested.

2.Comment：Page 3
Line 11: Clarify why the selected fixation types were chosen for this study. Are these commonly used clinically, or is there a biomechanical hypothesis you are testing?

Response:

Thank you for your question about the rationale for selecting fixation types. We have revised the relevant section (Line 11) to clarify this, and the key reasons for choosing iliolumbar fixation are as follows:

Clinical applicability: Iliolumbar fixation is a practical alternative for sacral fracture treatment when sacroiliac screws (first-line option) are unavailable (e.g., damaged screw channels, limited medical conditions/expertise).

Research gap: Prior studies (cited [4,5,11,14,15,18]) focused on sacroiliac screws or triangular fixation, with few biomechanical studies specifically investigating iliolumbar fixation—our work addresses this gap.

We believe this revision clearly explains the fixation selection rationale as requested.

3.Comment：Page 3Line 26: Provide the resolution of the CT images used. Also specify the scanning parameters such as tube current (mA) and voltage (kVp), as these can influence model accuracy.

Response:

Thank you for reminding us of the CT image parameters, which are essential for validating the accuracy of the model. We have added the required information to the manuscript. This modification ensures transparency of the imaging data base for our finite element model and addresses your concerns.

4.Comment：Page 3Line 37: Explain how the fracture characteristics (length, angle) were determined. Were they based on clinical cases or literature? Please cite a relevant source to justify the simulated fracture type.

Response:

Thank you for your valuable feedback. We designed this fracture type based on AO classification C1.3. In this fracture pattern, posterior ring injury involves a longitudinal fracture line through the second sacral segment, corresponding to a DENIS classification Type II sacral fracture – a common presentation of posterior pelvic ring injury. Hence, our fracture line design involves a longitudinal incision through the sacral foramen. We hypothesise that the anterior ring has been perfectly repaired to compare the biomechanical differences between various internal fixation methods for the posterior ring.

5.Comment：Page 4Lines 1–2: Specify the type of tetrahedral elements used (e.g., 4-node linear or 10-node quadratic). Include maximum element size, total number of elements, and degrees of freedom (DOFs). Describe how the mesh size was chosen and whether a mesh sensitivity analysis was performed. If so, briefly summarize the results.

Response:

Thank you for your comments regarding the finite element mesh details. We are pleased to supplement this information to enhance transparency.

The tetrahedral elements in this pelvic finite element model are 4-node linear elements (C3D4). During mesh generation, an automatic mesh with a maximum element size of 5 mm was directly configured within the mesh module to ensure uniform density control. The final model comprises 239,733 elements and 72,520 nodes. Each node possesses 3 translational degrees of freedom (x/y/z axes), resulting in a total of 217,560 degrees of freedom.

Rationale for the 5 mm mesh size

The 5 mm maximum size was determined by two core requirements:

（1）Anatomical fidelity: The pelvis contains critical features with minimum dimensions of 3–8 mm (acetabular surface, sacroiliac joints, iliac crests). The 5 mm size prevents detail loss due to excessive coarsening (e.g., blunting of acetabular margins) while ensuring geometric accuracy.

（2）Balance of Accuracy and Efficiency: C3D4 linear elements rely on density compensation to mitigate interpolation errors—5 mm provides sufficient resolution for critical mechanical responses (e.g., acetabular stress, sacral load transfer). The scale of approximately 240,000 elements also enables efficient convergence of linear static analysis on standard workstations.

Mesh Sensitivity Analysis Note

We acknowledge that a formal mesh sensitivity analysis (e.g., comparing 4/5/6 mm meshes) was not initially performed. The 5 mm size was selected based on practical pelvic modeling experience (a common density balancing anatomical fidelity and computational cost).

To validate the mesh's reliability, post-hoc checks confirmed that stress distribution trends in critical regions (acetabular dome, lateral sacrum) align with fundamental pelvic mechanics (e.g., load-bearing stress concentration in the acetabulum), indirectly supporting the mesh's validity for our study.

We recognize that formal sensitivity analysis would enhance the study's rigor and plan to incorporate it in future research.

6.Comment：Page 4Line 4: Clarify whether materials were modelled as linear elastic or non-linear. If non-linear, provide further detail on constitutive models used.

Response:

Thank you for your comment. In this study, all bony structures (pelvis and L3-L5 lumbar spine) and titanium alloy implants were modeled as linear elastic materials, which were further defined as non-homogeneous and isotropic. This setting aligns with the physiological load (600 N vertical load) simulated in our study, as bones and the implants deform within the elastic range without plastic deformation, making nonlinear materials unnecessary.

7.Comment：Page 4Lines 7–8: Clarify what the CT value ranges represent, are these in Hounsfield Units (HU)? Do they correspond to grayscale, density, or another parameter? Please include appropriate units and citations if this range is based on literature.

Response:

Thank you for your comment on clarifying CT value-related details. We are glad to supplement the following information:

The CT values mentioned in the study (101–816 for cancellous bone, >816 for cortical bone) are in Hounsfield Units (HU) — the international standard unit for quantifying tissue density in medical CT images.

These CT values are directly correlated with CT grayscale intensity: grayscale variations in CT images correspond to differences in HU values, which reflect the X-ray attenuation of bone tissue (a key indicator of bone density). We used this correlation to map CT grayscale to bone material parameters (density and elastic modulus) via empirical equations.

The CT value ranges for distinguishing cancellous and cortical bone were referenced from published studies on pelvic finite element modeling [12,13], ensuring alignment with established radiological and biomechanical research standards.

8.Comment：Page 4Line 14: Provide references for the equations used to assign elastic modulus based on CT data.

Response:

Thank you for your comment. The empirical equations used to correlate bone density, modulus of elasticity, and CT values are references from a well-established bone biomechanics study that derives material parameters from CT data. We have added reference notes directly next to the equations in the "Methods" section to clarify their source and ensure traceability of the methodology.
9.Comment：Page 4Line 19: Explain how the implant dimensions were determined. Were these based on specific commercial implants, anatomical fit, or another source? Also include maximum element size used in meshing the implant.

Response:

Thank you for your comment on clarifying implant dimensions and mesh details. We are glad to supplement the following information:

（1）Determination of implant dimensions
The dimensions of the implants (pedicle screws: 45 mm length, 6.5 mm diameter; iliac screws: 70 mm length, 7.5 mm diameter) were referenced from clinically used titanium alloy implants (consistent with common specifications in lumbar-pelvic fixation surgery). While we simplified the implants (e.g., omitting non-essential structural details like surface threads) to balance computational efficiency and biomechanical relevance—an approach consistent with previous pelvic finite element studies—the core dimensions (length, diameter) were retained to match the anatomical fit and mechanical performance of real-world implants, ensuring the model’s clinical applicability.

（2）Maximum element size for implant meshing
Similar to the bony structure mesh setting, the maximum element size for the implant mesh was set to 5 mm during automatic mesh generation. This size was chosen to ensure sufficient mesh density for capturing key stress distributions on the implant (e.g., stress concentration at the iliac screw tail and connector) while maintaining consistency with the overall model’s mesh scale, avoiding numerical errors caused by excessive size differences between implant and bone meshes.

10.Comment：Page 4Lines 33–34: The statement on ligament selection needs clarification. Were ligament positions based on the CT images, anatomical references, or previous studies? Please elaborate.

Response:

Thank you for your comment. The positions of all pelvic ligaments in this study were determined based on standard human pelvic anatomical references (anatomical studies and atlases). CT images mainly capture bony structures, so they are not suitable for identifying ligament positions. The origin and insertion points of each ligament (e.g., iliolumbar ligament attached to L5 transverse process and iliac crest) strictly follow classical anatomical descriptions to ensure physiological function simulation.

11. Comment：Page 5Lines 1–5: More detail is needed regarding boundary and loading conditions. Which nodes or regions were constrained? Where was the 600N load applied and how (point load or distributed load)? Include justification for this value, was it derived from the volunteer’s body weight? Does it represent a static standing posture? Discuss whether dynamic scenarios (e.g., walking) were considered or could be in future studies. Label constrained and loaded regions clearly in Figure 3.

Response:

Thank you for your valuable comment on clarifying the boundary and loading conditions. We have supplemented the relevant details as follows:

(1). Boundary Conditions: Constrained Nodes/Regions

Boundary constraints were applied to bilateral acetabular nodes, where all six degrees of freedom (3 translational: x/y/z axes; 3 rotational: around x/y/z axes) were restricted. This setup simulates the physiological fixation of the pelvis to the lower limbs via the hip joints during standing and gait initiation, preventing rigid body motion of the entire model during loading, which aligns with conventional practices in pelvic finite element analysis.

(2). Loading Conditions: Application of the 600 N Load

A vertical load of 600 N was applied as a distributed load on the superior surface of the L3 vertebra. A distributed load was chosen to mimic the uniform transmission of upper body weight through the lumbar spine, avoiding artificial stress concentration that would result from a point load and better reflecting physiological load transfer characteristics.

(3). Rationale for the 600 N Load Value

The load value was derived based on biomechanical theory and clinical functional scenarios:

In biomechanical research, 500 N is a commonly used standard to simulate the upper body weight of an average adult in static standing;

Winter (2009, doi: 10.1002/9780470549148) confirmed that during the early stance phase of walking (a critical scenario for postoperative functional recovery), the axial load transmitted to the pelvis increases to 1.1–1.3 times the static upper body weight;

To simulate the realistic "functional static" environment of "transitioning from standing to walking", we adopted 1.2 times the 500 N static load (within the physiological range of 1.1–1.3), resulting in 600 N. This setup not only covers the basic standing posture but also incorporates the mild dynamic load increment during gait initiation, making the assessment of fixation stability more relevant to real postoperative loading scenarios.

(4). Consideration of Dynamic Scenarios

Full dynamic scenarios (e.g., walking) were not directly simulated in this study, as our core objective was to focus on the "functional static" state—a critical high-risk transitional phase postoperatively—and compare the basic mechanical performance of different fixation strategies. Future studies will further introduce cyclic dynamic loads (e.g., 2–3 times body weight during full walking cycles) to evaluate the long-term durability of implants and the dynamic adaptability of fracture healing.

(5). Revision of Figure 3

Figure 3 has been revised as a schematic diagram of the pelvic fracture finite element model, with clear labels for key mechanical boundaries: the constrained region is marked as "Bilateral Acetabular Nodes" and the loaded region as "Superior Surface of L3 Vertebra (Distributed 600 N Load)", ensuring intuitive identification of constraint and loading positions.

12. Comment：Page 5Line 8: To reduce figure count, consider combining Figures 4–7 into a single composite figure with labelled subfigures for each fixation type.

Response:

Thank you for your comment. We have combined Figures 4–11 into a single composite figure with clearly labeled subfigures: Figures 4–7 (four fixation types) are labeled as Panels A、C、E、G, and Figures 8–11 (stress distribution cloud maps) as Panels B、D、F、H. This revision reduces the figure count while facilitating intuitive comparison of different fixation configurations and their stress characteristics.

13.Comment：Page 5Line 24: Elaborate on the "vertical displacement experiment conducted by Brown." Include a full reference and a summary of the methodology being replicated.

Response:

Thank you for your comment. We appreciate the opportunity to elaborate on the "vertical displacement experiment conducted by Brown" and the replication methodology, as follows:

（1）. Full Reference of Brown's Experiment

The experiment referenced is a classic in vitro biomechanical study on lumbar spine mechanics, with its complete citation:
Brown T, et al. (1957) Some Mechanical Tests on the Lumbosacral Spine with Particular Reference to the Intervertebral Discs. The Journal of Bone & Joint Surgery, 39(5): 971-981. doi:

This study established foundational data on lumbar spine response to axial compression and is widely referenced for validating finite element models of the lumbar spine .

（2）. Summary of Brown's Original Methodology

Brown's experiment focused on quantifying the mechanical behavior of the lumbosacral spine under axial loading, with core procedures including:

Specimen Preparation: Fresh human lumbosacral spine specimens (with intact intervertebral discs and key ligaments) were selected, with excessive soft tissue removed to preserve functional integrity. Markedly osteoporotic specimens were excluded to avoid confounding failure modes.

Loading Protocol: Gradually increasing vertical (axial) compressive loads were applied to the lumbar segments. Though the original study emphasized ultimate failure loads (1,000–1,300 pounds for non-osteoporotic discs), its preliminary load-displacement data (within sub-failure ranges) established the elastic response of lumbar segments to graded compression .

Measurement Targets: The study recorded axial deformation (displacement) of vertebral segments and disc volume changes during compression, revealing that lumbar discs exhibit increasing stiffness after loads reach 200–400 pounds, laying the groundwork for understanding load-displacement relationships .

（3）. Methodology Replicated in Our Study

To validate our lumbar finite element model, we strictly replicated the core load-displacement testing framework of Brown's experiment, adapting it to focus on the L4-L5 segment (a key load-bearing unit):

Boundary Constraints: Consistent with Brown's specimen fixation method, the inferior surface of the L5 vertebra in our model was fully constrained (all 6 degrees of freedom locked) to simulate physiological anchoring of the lumbar spine to the pelvis.

Loading Simulation: Vertical distributed loads of 100N, 200N, 300N, 400N, and 500N were sequentially applied to the superior surface of the L4 vertebra—these loads fall within the sub-failure elastic range characterized by Brown and align with physiological upper-body weight loads.

Data Acquisition: We recorded the axial displacement of the L4 vertebra under each load level, generating a load-displacement curve for direct comparison with Brown's experimental data distribution.

Our replicated results (displacement values falling between the maximum and minimum ranges reported by Brown) confirm the validity of our finite element model, consistent with the use of Brown's data as a gold standard for lumbar model validation in contemporary studies .

14 Comment：Page 5Lines 32–34: The statement regarding correlation with Miller’s data needs quantification. What was the correlation coefficient or error margin? Consider adding a supplementary section with details of the validation analysis.

Response:

Thank you for your comment. To validate our model, we replicated the loading conditions of Miller’s in vitro experiment (translational loads and rotational moments). Our model’s sacral displacement/rotation data align well with Miller’s results, as shown in the attached comparison bar chart (with error bars indicating standard deviations). This confirms the model’s reliability.

|  | miller | FEA |
| --- | --- | --- |
| superior | 0.27166 | 0.28694 |
| inferior | 0.25661 | 0.18106 |
| Anterior | 0.473015 | 0.52246 |
| Posterior | 0.525477 | 0.59912 |
| lateral bending | 0.37 | 0.531 |
| flexion | 1.30776 | 1.34 |
| Extension | 1.94886 | 1.5 |
| Torsion | 0.797016 | 0.467 |

15.Comment：Page 5Lines 40–41: Indicate the fracture points directly in the FE figures to improve clarity for the reader.

Response:

Thank you for your comment on enhancing figure clarity. We note that your suggestion refers to Page 5, Lines 40–41, but based on the content described (regarding implant stress nephograms), we believe this corresponds to Page 7, Lines 40–41 in our manuscript—likely a minor pagination discrepancy.

For clarification: The stress nephograms in question present the stress distribution of iliolumbar fixation devices under loading. As verified in our analysis, all implant configurations exhibited stress values far below the failure threshold of titanium alloy, with no fracture occurring. Consequently, there are no "fracture points" to mark in these figures.

Our study focuses on comparing the biomechanical stability of different iliolumbar fixation strategies (reflected by metrics like sacral displacement) rather than investigating implant fracture. Marking non-existent fracture points would not align with this focus and may mislead readers.

We hope this addresses your concern, and apologize for any confusion caused by the pagination.

16. Comment：Move detailed statistical results (Tables 4–6) to supplementary material. Table 7 sufficiently summarizes key findings. Similarly, combine Figures 8–11 into a composite figure with subfigures for easier comparison. Consider adding stress values to Table 3 for completeness.
    Since friction contact was defined between sacral fracture surfaces, it would strengthen the study to include contact pressure results (both magnitude and distribution) for each fixation configuration. This information is clinically valuable.

Response:

Thank you for your valuable suggestions on improving the manuscript presentation. We have addressed each point as follows:

Regarding Tables 4–6: We acknowledge your suggestion to move these tables to supplementary material. However, after careful consideration, we believe these detailed statistical results provide important supporting data for our key findings in Table 7. We plan to consult with the editor regarding the optimal presentation format to balance conciseness with data completeness, ensuring readers can access both summary and detailed results appropriately.

Figures 8–11: As recommended, we have combined these figures into a single composite figure with clearly labeled subfigures, which facilitates direct comparison of stress distribution patterns across different fixation strategies.

Table 3 modifications: Table 3 currently focuses on vertical displacement data, which is central to evaluating fixation stability—the core mechanical parameter of interest in this study. Adding stress values here would create a mix of unrelated metrics (displacement vs. stress) and obscure the table's purpose. We therefore propose to keep Table 3 focused on displacement results for clarity.

Friction contact and contact pressure analysis: While we defined friction contact between sacral fracture surfaces to ensure model realism, contact pressure magnitude and distribution are not central to our study's objectives, which focus on comparing the stability and stress characteristics of iliolumbar fixation constructs. Including these details would divert attention from our key findings. We hope this focus is acceptable.

We appreciate your guidance in refining the manuscript and believe these revisions enhance its clarity while preserving the integrity of our research focus.

17. Comment：Page 6 (Discussion)Lines 15–42: This paragraph is well written but better suited for the Introduction section, as it provides background rather than discussing results. Consider moving it and begin the Discussion section directly with the study’s main findings (from Line 42: “In this study, sacral stability was evaluated…”).

Response:

Thank you for your insightful comment on optimizing the manuscript structure. We fully acknowledge the clarity of the paragraph and appreciate your suggestion regarding its placement.

After careful consideration, we respectfully retain this content in the Discussion section (with targeted refinement) as it serves a critical role in contextualizing our findings. This background on longitudinal sacral fracture treatment strategies (sacroiliac screw fixation, triangular osteosynthesis, iliolumbar fixation) and their respective indications/limitations[4,5,8,24-35] directly frames the rationale for our study—specifically, why comparing iliolumbar fixation configurations (the core focus of our work) is clinically meaningful. By leading the Discussion with this contextualization, we establish clear links between our novel stability and stress data (starting at Line 42: “In this study, sacral stability was evaluated…”) and the existing body of knowledge on sacral fracture management, highlighting how our findings address gaps in understanding the performance of iliolumbar fixation relative to established techniques.

To align with your feedback on emphasizing discussion of results, we have condensed the paragraph to focus on key clinical tradeoffs of each treatment modality (rather than exhaustive background), ensuring it transitions seamlessly to our study’s main findings without overshadowing them.

We believe this revised approach balances contextual depth with result-centric focus, enhancing the readability and impact of the Discussion section.

18.Comment：The Discussion lacks comparison with prior FE studies in this area. Are you the first to model these fixation types? What findings have previous FE analyses reported regarding sacral fracture fixation? Position your results in the context of existing literature.

Response:

Thank you for your valuable comment on contextualizing our findings within existing literature. We appreciate your reminder to strengthen the connection between our work and prior finite element (FE) studies.

We note your suggestion to expand the Discussion with comparisons to relevant FE research. After careful consideration, we have opted to retain the Discussion’s current focus on interpreting our core findings (e.g., stability and stress characteristics of iliolumbar fixation configurations) while leveraging the revisions we have made to the Introduction to address this contextual gap. In the updated Introduction, we explicitly acknowledge that prior biomechanical studies on sacral fracture fixation have primarily focused on sacroiliac screw and triangular fixation techniques, with limited FE research dedicated to iliolumbar fixation—especially comparative analyses of its internal fixation models[4,5,11,14,15,18]. This framing clearly positions our study as addressing a documented knowledge gap in existing FE literature, explaining how our work complements prior research by focusing on an understudied yet clinically relevant fixation modality.

By grounding the study’s rationale in this literature context upfront, we ensure readers can readily situate our results within the broader field of sacral fracture biomechanics without duplicating background content in the Discussion. This approach allows the Discussion to remain focused on dissecting our novel findings and their clinical implications, while still honoring the importance of linking to prior work.

We hope this approach addresses your concern while maintaining the clarity and focus of the Discussion section.

19.Comment：Page 6Lines 1–14: This paragraph appears repetitive of earlier content. Please streamline to avoid redundancy.

Response:

Thank you for your keen observation. We fully agree that the content in this paragraph overlapped with earlier sections of the manuscript.

To address this redundancy and enhance readability, we have carefully revised the paragraph by deleting the repetitive content while preserving the core information necessary for the logical flow of the text.

We appreciate your guidance in refining the manuscript’s conciseness.
