# Supplementary material for: Biomechanical optimization of iliolumbar fixation strategies for unilateral vertical sacral fractures: Prioritizing stability-mobility balance via finite element analysis
Source: PLoS One. 2026 Jan 13;21(1):e0339705. doi: 10.1371/journal.pone.0339705 (PMC12798994; doi:10.1371/journal.pone.0339705)
Supplement: Attachment — Submitted filename: Response to Reviewer.docx [file pone.0339705.s007.docx]

Response to Reviewer#3 Comments

1.Comment :
The authors justify mesh density through an “adaptive meshing strategy” but do not provide quantitative convergence evidence. A simple sensitivity graph (e.g., maximum sacral displacement vs. element size) can be included if possible.

Response:

We thank the reviewer for highlighting the importance of mesh-convergence evidence. Our study was designed to compare fixation strategies rather than to develop a new FE methodology, and we therefore adopted a standardized meshing protocol (4-node linear tetrahedra, maximum element size 5 mm) that lies within the range commonly reported for pelvic FE models and validations in the literature (e.g., Refs. [11–13, 21] of our manuscript). In the present revision we have clarified the meshing rationale in the Methods and, to avoid over-stating precision, we explicitly acknowledge as a limitation that we did not include a formal displacement-versus-element-size convergence plot. We will address this with a full parametric convergence study in subsequent work focused on methodological aspects.

2.Comment :
Verification and validation should be distinguished. Verification requires mesh quality and solver stability checks; validation requires comparison with experimental or published benchmark data. Currently these are conflated.

Response:

We thank the reviewer for highlighting the distinction between verification and validation. We acknowledge that, in the original submission, these concepts were presented together. In line with the reviewer’s guidance—and without extending the scope beyond our current resources—we have revised the manuscript to clearly separate the two concepts and to state explicitly the limits of what we performed. Specifically, we did not carry out a formal mesh‐quality audit or a dedicated solver‐stability study. Our comparative analysis therefore relies on a standardized meshing protocol (C3D4; 5-mm maximum element size) and identical modeling assumptions across all constructs to ensure fair between-construct comparisons.
For validation, we kept literature-based, range/trend comparisons with published experimental/benchmark data (Brown/Markolf for the spine; Miller for the pelvis/SI joint) to establish physiological plausibility. These clarifications have been added to Methods and Limitations. A protocol-matched quantitative calibration and a full numerical verification campaign are valuable but beyond the scope of the present comparative study.

3.Comment : Material properties and load magnitudes are taken from the literature, which is acceptable. Nonetheless, even a limited sensitivity check (e.g., ±10% variation in elastic modulus of bone) would strengthen confidence in the results.

Response:

We appreciate the reviewer’s suggestion to include a ±10% material-property sensitivity. We agree that such an analysis can further strengthen confidence. At present, however, our study is scoped as a comparative biomechanics analysis rather than a methodological parametric study, and adding new simulation batches would substantially expand the workload beyond our current timeline. In the revision, we therefore (i) clarify that bone and soft-tissue properties and loads were selected from well-cited pelvic FE literature, (ii) explain that, under our small-strain linear-elastic assumptions, uniform ±10% perturbations to bone elastic modulus largely scale absolute displacements/stresses without altering the relative ordering between fixation constructs, and (iii) acknowledge in the Limitations that a formal multi-parameter sensitivity analysis is valuable and will be pursued in subsequent work.

4.Comment : While the Brown and Miller experimental studies are cited, the validation is qualitative. Quantitative comparison (correlation coefficients, error margins, RMSE) should be added to substantiate model accuracy.

Response:

We thank the reviewer for encouraging a quantitative validation (e.g., correlation, error margins, RMSE). We agree that such metrics are valuable for methodological calibration studies. Our work, however, was scoped as a comparative biomechanics analysis of fixation constructs rather than a full calibration of an FE pelvis model. Consistent with the validation practice in prior pelvic FE literature we cited, we adopted literature-based, trend-level validation against Brown/Markolf (spine) and Miller (pelvis/SI joint) to establish physiological plausibility (i.e., responses falling within reported ranges and reproducing stiffness/ordering trends). A direct computation of correlation/RMSE was not feasible here because the published datasets do not provide specimen-matched raw curves under identical boundary conditions and contact/friction assumptions; mixing heterogeneous set-ups can yield misleading statistics.

In the revision, we (i) clarify the verification–validation distinction, (ii) state explicitly that our validation is range-/trend-based due to the above constraints, and (iii) add a Limitation noting that a formal quantitative calibration (with matched protocols and raw data access) is beyond the scope of this comparative study but is a valuable direction for future work. We hope these clarifications address the concern without altering the study’s original scope.

5.Comment : Stress contour plots require scale bars (MPa), clear colour legends, and loading direction indicators. Tables should fully comply with SI units and consistent significant figures (e.g., 2–3 s.f.). Figure captions should be descriptive enough for standalone interpretation.

Response:

We appreciate the reviewer’s guidance on figure annotation and SI reporting. In this study, stress maps are included as supporting evidence to show that peak implant stresses remain below the titanium alloy yield strength, while the primary outcomes are sacral displacement and construct stability. To improve clarity without expanding the figure footprint, we revised the composite stress figure by adding a colour-bar label in MPa (von Mises stress). Because von Mises stress is a scalar, explicit direction arrows are not required for interpreting peak levels; the uniform loading/boundary conditions (600 N vertical distributed load at L3; bilateral acetabula constrained) are stated in the caption and Methods.
Regarding SI units and significant figures, the manuscript already uses SI units throughout. For numerical precision, we have retained the original computational precision in certain tables (occasionally 4 decimals) to preserve the integrity and reproducibility of the completed statistical analyses (ANOVA/LSD, normality and variance tests). Ex-post rounding would require reformatting outputs and could introduce minor inconsistencies with the reported statistics. We hope these concise updates address the comment while preserving a clean layout and statistical transparency.

6. Comment :The Discussion repeats some results and could be more concise. The clinical perspective has been improved but should also discuss potential long-term risks such as fatigue failure, implant loosening, and patient-specific variability. Prior FE studies on sacral fracture fixation should be explicitly compared, beyond general references.

Response:

We thank the reviewer for these helpful suggestions. In the revision we have streamlined the Discussion and focused it on interpretation and clinical decision-making for iliolumbar segment selection, rather than restating numerical outputs.Concretely:Removed repetition of detailed means/p-values and rephrased the opening to present the clinical trade-off between stability and lumbar-motion preservation.Clarified the construct-level message: under identical conditions, dual-segment iliolumbar constructs (L4+L5+IS; L5+S1+IS) reduced sacral vertical displacement relative to single-segment options, while all peak implant stresses remained below titanium yield; L5+S1+IS and L4+L5+IS offered comparable stability with different implications for motion preservation.Added a focused paragraph on long-term risks: potential fatigue under cyclic loading, risk of implant loosening at high-stress interfaces (e.g., iliac screw connector), and patient-specific variability (BMD, sacral morphology). We also provide rehabilitation recommendations (graduated weight-bearing and core stabilization) when short-segment constructs are selected.Provided an explicit comparison with prior FE literature: most prior work centers on sacroiliac screws or triangular/combined constructs; our findings add iliolumbar-specific evidence by isolating the effect of segment/level selection, complementing SI-screw-centric analyses that vary screw length/trajectory.

These edits make the Discussion more concise and clinically interpretable without changing the study scope or adding new simulations. The revised text appears in Discussion, revised paragraphs.

7. Comment :The authors acknowledge missing patient-specific parameters and lack of dynamic loading. These limitations should be expanded, emphasising their impact on clinical extrapolation.

Response:

We thank the reviewer for this important point. In the revision, we expanded the Limitations to explicitly discuss (i) the absence of patient-specific parameters (e.g., bone mineral density [BMD], sacral morphology, ligament properties, implant–bone interface conditions) and (ii) the lack of dynamic/cyclic loading. We now state that these factors may affect absolute magnitudes (displacement/stress) and the fatigue/loosening risk profile, thereby limiting clinical extrapolation to populations whose anatomy and bone quality are close to our modeling assumptions. We emphasize that our conclusions should be interpreted as relative, between-construct comparisons under uniform assumptions, and are most applicable to adult patients without severe osteoporosis or highly comminuted/bilateral injuries.
We also outline future work: subject-specific FE with BMD-mapped material properties, probabilistic sensitivity to patient variability, cyclic loading (10^5–10^6 cycles) for fatigue safety margins, and more realistic implant–bone/contact models (frictional or cohesive behavior). These additions clarify the scope and the boundaries of clinical translation without changing the study’s comparative focus.

8 .Comment :Standardise terminology throughout (iliolumbar fixation vs. spino-pelvic fixation).

Response:

We appreciate the reviewer’s suggestion to standardise terminology. In the revised manuscript we consistently use “iliolumbar fixation” for the surgical technique. At its first mention, we add a note that prior literature may refer to the same construct as lumbopelvic/spinopelvic fixation; thereafter, the text, figures, tables, and captions uniformly use iliolumbar fixation. We retain lumbopelvic only when describing the anatomical load-transfer region or when citing the original titles of references. In Keywords, to avoid duplication we replaced “Spino-pelvic fixation” with “Iliac screw”, which better reflects the constructs analysed and improves indexing.

9 .Comment :Proofread carefully for residual grammatical inconsistencies.
Response:

Thank you for the reminder. We have carefully proofread the manuscript and corrected residual grammatical and formatting issues. Terminology is now consistent (iliolumbar fixation), SI units and p-value formatting are unified (e.g., 600 N; 259.4 MPa; p < 0.001), and figure/table citations and minor typos have been fixed. These edits do not affect the scientific content or results.
