# Supplementary material for: Biomechanical optimization of iliolumbar fixation strategies for unilateral vertical sacral fractures: Prioritizing stability-mobility balance via finite element analysis
Source: PLoS One. 2026 Jan 13;21(1):e0339705. doi: 10.1371/journal.pone.0339705 (PMC12798994; doi:10.1371/journal.pone.0339705)
Supplement: Attachment — Submitted filename: Response_to_Reviewer_auresp_3.docx [file pone.0339705.s008.docx]

Response to Reviewer#3 Comments

1.Comment :
The concept of biomechanical optimisation should be explained more clearly — is there a description of the optimisation procedure or workflow?
Some charts and FEA printouts are not sufficiently clear; the legends and units should be fully legible to ensure readability.

Response:

Thank you for your valuable comments. We have addressed all your concerns with concise revisions, as detailed below:

Regarding the query on "biomechanical optimization": We clarify that "biomechanical optimization" in this study refers to comparing the stability, implant stress, and lumbar mobility of four iliolumbar fixation models via finite element analysis, to identify the optimal clinical strategy for different scenarios. Corresponding revisions have been made:

Title: Revised to "Biomechanical optimization of iliolumbar fixation strategies for unilateral vertical sacral fractures: prioritizing stability-mobility balance via finite element analysis" (the addition of "prioritizing stability-mobility balance" clarifies the optimization core).

Abstract Objective: Revised to "This study aims to optimize iliolumbar fixation strategies for unilateral vertical sacral fractures via finite element analysis, by comparing stability, implant stress, and lumbar mobility to identify the optimal clinical option" (articulates the optimization logic).

Regarding the clarity of figures and finite element analysis outputs: We have revised the relevant figures to enhance readability, ensuring all legends and units are fully distinguishable.

The core content of the study remains unchanged. We believe these revisions fully address your concerns. Thank you again for your careful guidance!
